# High-Quality Exome Sequencing of Whole-Genome Amplified Neonatal Dried Blood Spot DNA

**DOI:** 10.1371/journal.pone.0153253

**Published:** 2016-04-18

**Authors:** Jesper Buchhave Poulsen, Francesco Lescai, Jakob Grove, Marie Bækvad-Hansen, Michael Christiansen, Christian Munch Hagen, Julian Maller, Christine Stevens, Shenting Li, Qibin Li, Jihua Sun, Jun Wang, Merete Nordentoft, Thomas Mears Werge, Preben Bo Mortensen, Anders Dupont Børglum, Mark Daly, David Michael Hougaard, Jonas Bybjerg-Grauholm, Mads Vilhelm Hollegaard

**Affiliations:** 1 Department for Congenital Disorders, Danish Centre for Neonatal Screening, Section of Neonatal Genetics, Statens Serum Institut, Copenhagen, Denmark; 2 Department of Biomedicine, Aarhus University, Aarhus, Denmark; 3 iPSYCH - Lundbeck Foundation Initiative for Integrative Psychiatric Research, Aarhus University, Aarhus, Denmark; 4 iSEQ - Centre for Integrative Sequencing, Aarhus University, Aarhus, Denmark; 5 Bioinformatics Research Centre, Aarhus University, Aarhus, Denmark; 6 Department for Congenital Disorders, Molecular Medicine, Statens Serum Institut, Copenhagen, Denmark; 7 Broad Institute, Stanley Center, Cambridge, Massachusetts, United States of America; 8 BGI-Shenzhen, Shenzhen, China; 9 BGI-Europe A/S, Copenhagen, Denmark; 10 Mental Health Centre Copenhagen, Faculty of Health Sciences, University of Copenhagen, Copenhagen, Denmark; 11 Mental Health Centre Sct. Hans, Institute for Biological Psychiatry, Capital Region of Denmark, Roskilde, Denmark; 12 National Centre for Register-based Research, School of Business and Social Sciences, Aarhus University, Aarhus, Denmark; 13 Department for Congenital Disorders, Danish Centre for Neonatal Screening, The Danish Neonatal Screening Biobank, Statens Serum Institut, Copenhagen, Denmark; Odense University Hospital, DENMARK

## Abstract

Stored neonatal dried blood spot (DBS) samples from neonatal screening programmes are a valuable diagnostic and research resource. Combined with information from national health registries they can be used in population-based studies of genetic diseases. DNA extracted from neonatal DBSs can be amplified to obtain micrograms of an otherwise limited resource, referred to as whole-genome amplified DNA (wgaDNA). Here we investigate the robustness of exome sequencing of wgaDNA of neonatal DBS samples. We conducted three pilot studies of seven, eight and seven subjects, respectively. For each subject we analysed a neonatal DBS sample and corresponding adult whole-blood (WB) reference sample. Different DNA sample types were prepared for each of the subjects. Pilot 1: wgaDNA of 2x3.2mm neonatal DBSs (DBS_2x3.2) and raw DNA extract of the WB reference sample (WB_ref). Pilot 2: DBS_2x3.2, WB_ref and a WB_ref replica sharing DNA extract with the WB_ref sample. Pilot 3: DBS_2x3.2, WB_ref, wgaDNA of 2x1.6 mm neonatal DBSs and wgaDNA of the WB reference sample. Following sequencing and data analysis, we compared pairwise variant calls to obtain a measure of similarity—the concordance rate. Concordance rates were slightly lower when comparing DBS vs WB sample types than for any two WB sample types of the same subject before filtering of the variant calls. The overall concordance rates were dependent on the variant type, with SNPs performing best. Post-filtering, the comparisons of DBS vs WB and WB vs WB sample types yielded similar concordance rates, with values close to 100%. WgaDNA of neonatal DBS samples performs with great accuracy and efficiency in exome sequencing. The wgaDNA performed similarly to matched high-quality reference—whole-blood DNA—based on concordance rates calculated from variant calls. No differences were observed substituting 2x3.2 with 2x1.6 mm discs, allowing for additional reduction of sample material in future projects.

## Introduction

The accurate genotyping of DNA of neonatal dried blood spot (DBS) samples has proven plausible, and opened new avenues in neonatal screening and for identification of determinants in complex genetic diseases [1, 2]. Several countries store the excess neonatal DBS samples in repositories known as biobanks, but in only a few of the cases the samples are stored at frost, including Denmark in which storage is at -20°C [3–8]. Frost storage generally serves to preserve the overall sample quality compared to samples stored at room temperature, which will be more restricted in their use. Combined with the relevant clinical information, the neonatal DBS samples can be used for genome-wide association studies of disease to provide for the necessary cohort size [9].

In Denmark neonatal DBS samples are stored in the Danish Neonatal Screening Biobank (DNSB), which today contains over two million samples representing close to 100% of the Danes born after 1982 [8, 10]. At birth, Danish citizens are assigned a unique person-identifying number used across all public registration systems including the DNSB and in the well-established public health care system. This makes it possible to study the entire population under a certain age as a single cohort, and consequently to identify the determinants of common and complex genetic diseases in the Caucasian ethnicity [11]. The use of neonatal DBS samples for such ventures is however, challenged by the very small amount of blood available with three spots of ~50 μl blood per sample being collected for neonatal screening. The majority of surplus material is often also reserved for other purposes, including in order of priority: analysis for the benefit of the child and family, development and optimization of the current screening programme and research projects [9]. Most projects are therefore limited to a maximum of two discs of 3.2 mm size per sample with each spot yielding a maximum of 60 ng of retrievable DNA [12]. This restricts the use of neonatal DBS samples to a limited set of technologies/platforms, most often with no possibility of repeating the experiments. For genetic studies this may be overcome by whole-genome amplification (WGA) of the DNA (wgaDNA) leading to microgram quantities [13]. The genotyping accuracy of neonatal wgaDNA has previously been questioned. However, several studies have shown that the wgaDNA performs equally well compared to high-quality DNA of other sources, using low- and high-throughput genotyping platforms [1, 14, 15]: the wgaDNA has been used with great success in research projects looking at infantile hypertrophic pyloric stenosis [16], birth weight [17], schizophrenia [18, 19], psychosis [20] and cerebral palsy [21]. We recently found that the wgaDNA also works for next-generation sequencing (NGS) [22]: an adult whole-blood (WB) reference sample (WB_ref), a three-year old DBS reference sample of the same person and the corresponding archived neonatal DBS sample were compared. The reference DBS and neonatal DBS samples were WGA following DNA extraction, which was not the case for the WB_ref. The data were of high quality containing similar SNP error rates for all sample types inspected, no matter the approach taken. However, as this study only included two subjects, the robustness’s of the methodology still needs to be confirmed.

This paper forms a continuation of the work done in Hollegaard et al. [22], where we seek to establish and expand the feasibility of using neonatal DBS samples for exome-targeted next-generation sequencing (WES). We aim to show that WGA is robust and reproducible; independent of kit and sequencing site. Thus, three different library preparation kits and two different sequencing sites were used for this study. The cohort size is expanded to include 22 subjects matched by a neonatal DBS sample and adult WB reference sample each. The study uses a comparison of replicated WB samples to establish a base-line of technical reproducibility, any variance beyond that of the WB replicas is inferred to be due to the WGA. The compatibility of using smaller inputs of neonatal DBSs (i.e. smaller disc size) with the sequencing technology is also investigated. This could provide for greater flexibility in future project designs and/or for neonatal screening. The relative sample performance is inferred from a similarity measure—the concordance rate—representing a pairwise comparison of genotypes calls at the variant sites. This allows us to compare the DBS-derived wgaDNA with high-quality DNA of a WB reference sample, to provide the final call of sample performance.

## Methods

### Study Design and Samples

This study represents a compilation of three separate pilot studies: Pilot 1, Pilot 2 and Pilot 3 containing seven, eight and seven subjects, respectively. We used a neonatal DBS sample and a corresponding adult WB reference sample for each individual, which were retrieved from the DNSB [23]. As defined by the 'Danish Act on Research Ethics Review of Health Research Projects' Section 2, this project does not constitute a health research project but is considered a quality developmental project for neonatal screening—thus it can be conducted without approval from the Committees on Biomedical Research Ethics for the Capital Region of Denmark.

**Fig 1:**
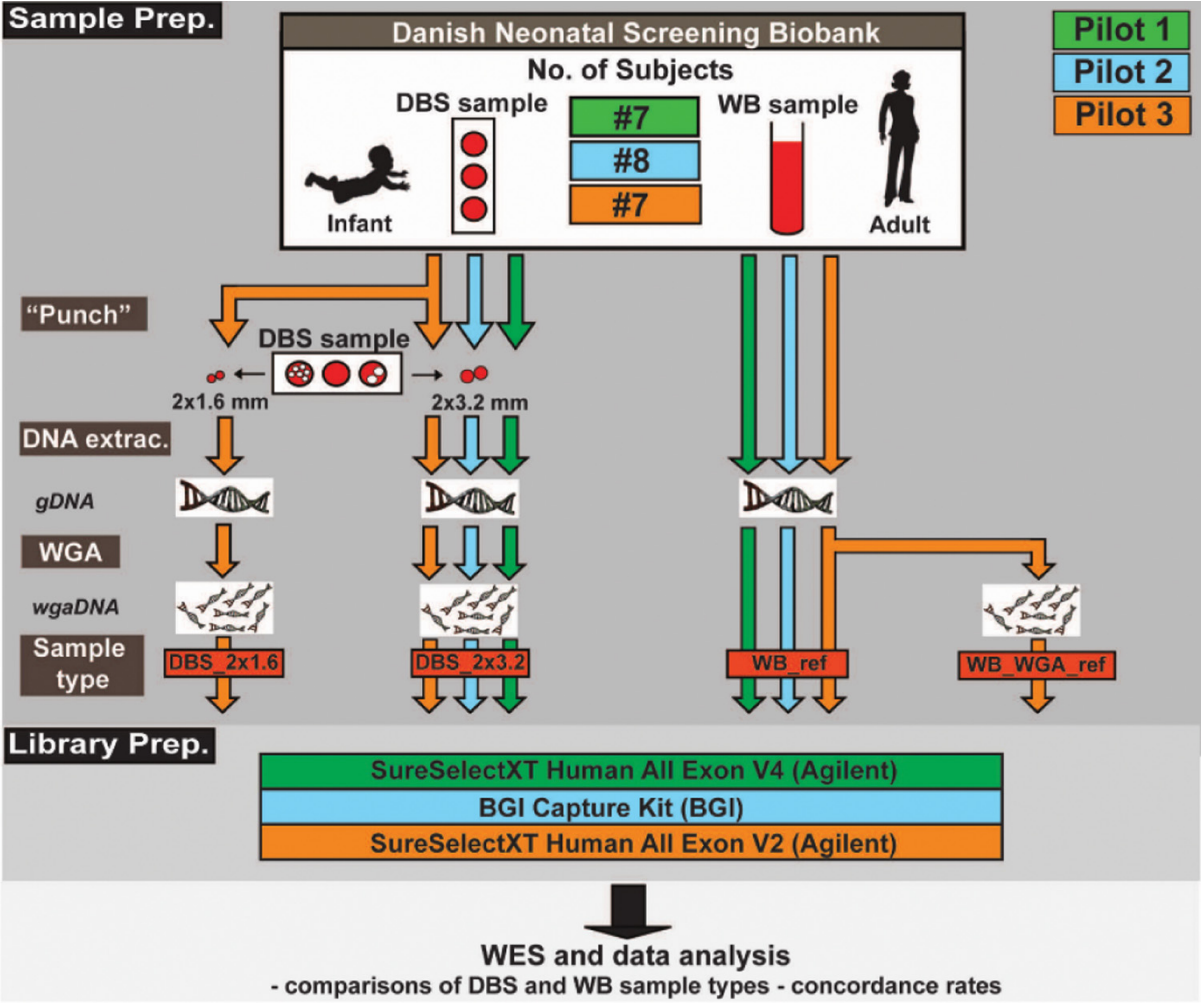
Study design. This study aims to identify systematic differences between DBS and WB by testing both sample types from a number of individuals. Pilot 1, Pilot 2 and Pilot 3 included seven, eight and seven subjects represented with a neonatal DBS sample and corresponding adult WB reference sample each, respectively. A minimum of two sample types per subject were prepared for library preparation: wgaDNA of DNA from 2x3.2 mm of neonatal DBSs (DBS_2×3.2) and raw control DNA from the WB reference sample (WB_ref). Pilot 2 also included a replicate sample of each of the WB_ref samples (WB_ref_replicate)(replicate sample not shown in the cartoon). Pilot 3 included two additional sample types; WGA of DNA from 2x1.6 mm neonatal DBS (DBS_2x1.6) and WGA of the WB reference sample (WB_WGA_ref). Note that the DBS_2x1.6 sample type of Pilot 3 was prepared and sequenced in triplicate using different sets of 2x1.6 mm discs for all of the subjects included. The samples were set up for library preparation using different kits in the respective pilot studies, before sequencing and data analysis. Please note the color-coding used with green, blue and orange specifying the respective pilot studies. All samples were retrieved from the Danish Neonatal Screening Biobank.

Fig 1 provides a detailed overview of the study design and sample preparation used in the respective pilots. Note that all details of sample preparation relevant to differentiate between DNA sample types are described herein. The sample types prepared were for Pilot 1: DBS_2×3.2 and WB_ref sample types; Pilot 2: DBS_2×3.2, WB_ref and WB_ref_replica sample types and Pilot 3: DBS_2×1.6, DBS_2×3.2, WB_WGA_ref and corresponding WB_ ref sample types. DNA extractions of the WB reference samples were with the Maxwell 16 LEV Blood DNA Kit (Promega) as prescribed by the manufacturer, and DNA extractions of the neonatal DBS samples were with the Extract-N-Amp Blood PCR Kit (Sigma) as described previously [1]. WGA was carried out in triplicate using the REPLIg Kit (QIAGEN) as has also been described previously [1]. The DNA was quantified using the Quant-IT PicoGreen dsDNA Reagent (Invitrogen) before library preparation and sequencing.

### Sequencing and Library Preparation

Library preparation and sequencing of the samples in Pilot 1 and Pilot 2 were performed at BGI-Europe, Copenhagen, Denmark—and those of Pilot 3 at Broad Institute's Genomics Platform, Cambridge, USA. The total number of samples processed were in Pilot 1: 14 (7 subjects of DBS_2x3.2 and WB_ref sample types), Pilot 2: 24 (8 subjects of DBS_2x3.2, WB_ref and WB_ref replica sample types) and Pilot 3: 42 (7 subjects of DBS_2x3.2, DBS_2x1.6 (prepared in triplicate), WB_ref and WB_WGA_ref sample types). Different library preparation kits were used in the pilots. Pilot 1: SureSelectXT Human All Exon V4 kit (Agilent), Pilot 2: a BGI Capture kit (produced in-house at BGI) and Pilot 3: SureSelectXT Human All Exon V2 kit (Agilent). The libraries of Pilot 1 and Pilot 3 were prepared according to the manufacturers instructions using 1 μg of input DNA per sample. Library preparation in Pilot 2 were with 500 ng of DNA as input per sample. Library validation was with the KAPA Library Quantification Kit (KAPA Biosystems), and sequencing was performed on a HiSeq2000 Sequencer for the samples of Pilot 1 and Pilot 2 and a HiSeq2500 Sequencer for the samples of Pilot 3.

### Data Analysis

#### Mapping

The sample reads were aligned to the genome (reference GRCh37) using BWA version 0.7.4, converted to BAM format and indexed using SAMtools (version 0.1.18). The samples were re-aligned, marked for duplicates and recalibrated using GATK and Queue (version 2.7–2) as pipeline manager.

*Variant Calling*: the variants were called using HaplotypeCaller and UnifiedGenotyper, processed with VQSR following the best practices for the version in use, and pre-filtered by 'PASS' at the output of each caller. The calls were merged by including, in order of priority: All 'PASS' HaplotypeCaller variants and All 'PASS' calls unique to UnifiedGenotyper. This implies that *i)* in case of overlapping 'PASS' variants, the calls from HaplotypeCaller were included, *ii)* in case of overlapping variants filtered by VQSR in HaplotypeCaller and 'PASS' in UnifiedGenotyper, the UnifiedGenotyper calls were included and *iii)* all 'PASS' non-overlapping variants unique to each caller were included.

#### Annotation

The variants were annotated using the snpEFF (version 3.3h), EPACTS (version 3.3) and Variant Effect Predictor (version 75) tools from ENSEMBL, and the variant type using the GATK VariantAnnotator. Based on this, the variant calls were grouped into SNPs, insertions, deletions and multiallelelic calls. The group of multiallelic calls comprises the variant types identified by GATK as 'MULTIALLELIC_COMPLEX.Other' and 'MULTIALLELIC_MIXED': the first includes the variants represented by multiple alleles containing insertions or deletions (or a combination hereof) of different sizes, while the second includes the variants in which multiple alternative alleles can be a combination of SNPs, insertions and/or deletions.

*Filtering*: both a variant-level approach (depth) and a sample-level approach (genotype quality) were employed for filtering of the variant calls: we set a threshold for the average sample depth >20 and for the minimum genotype quality (minGQ) across the WB_ref samples (minGQ_WB) >30. The average sample depth was calculated by dividing the multi sample depth with the number of samples included in the respective callings. The minGQ_WB criteria only considers the variant calls of the WB_ref samples using this as the high-quality standard. In case of missing data, the minGQ_WB value was set to 0.

#### Concordance

Sample performance was evaluated based on a pairwise comparison of genotypes (the variant calls) resulting in a similarity measure between any two samples—the concordance rate. Similarity measures were categorized in five groups as: *concordant*–for identical genotypes between samples the calls being either present or missing, *discordant (het/homo)*–for genotype calls differing between samples one call being homozygous (for the reference or for the first alternative allele) and the other heterozygous (reference, or first alternative allele), *discordant (homo/homo)*—for genotype calls differing between samples one being homozygous for the reference and the other homozygous for the first alternative allele, *discordant (other)*—for genotype calls differing between samples and which involve alternative alleles other than the first, *missing*–for genotypes exhibiting 'no call' in only one of the two samples. Here only the concordance rate has been used to highlight the key element of our performance comparisons: probing the relative similarity of wgaDNA of neonatal DBS samples to high-quality DNA of WB samples obtained from the same subjects. We have compared the paired samples, i.e. originating from the same subject of the DBS_2x3.2 and WB_ref sample types in all pilots (comparisons will henceforth be denoted as [sample type 1] vs [sample type 2]). In Pilot 2, we also compared the WB_ref and WB_ref replica sample types for comparison to the DBS_2x3.2 vs WB_ref comparisons. Additional comparisons made for Pilot 3 were DBS_2x1.6 vs WB_ref, DBS_2x3.2 vs DBS_2x1.6 and WB_ref vs WB_WGA_ref, respectively.

## Results and Discussion

### WES Statistics—Coverage

**Fig 2:**
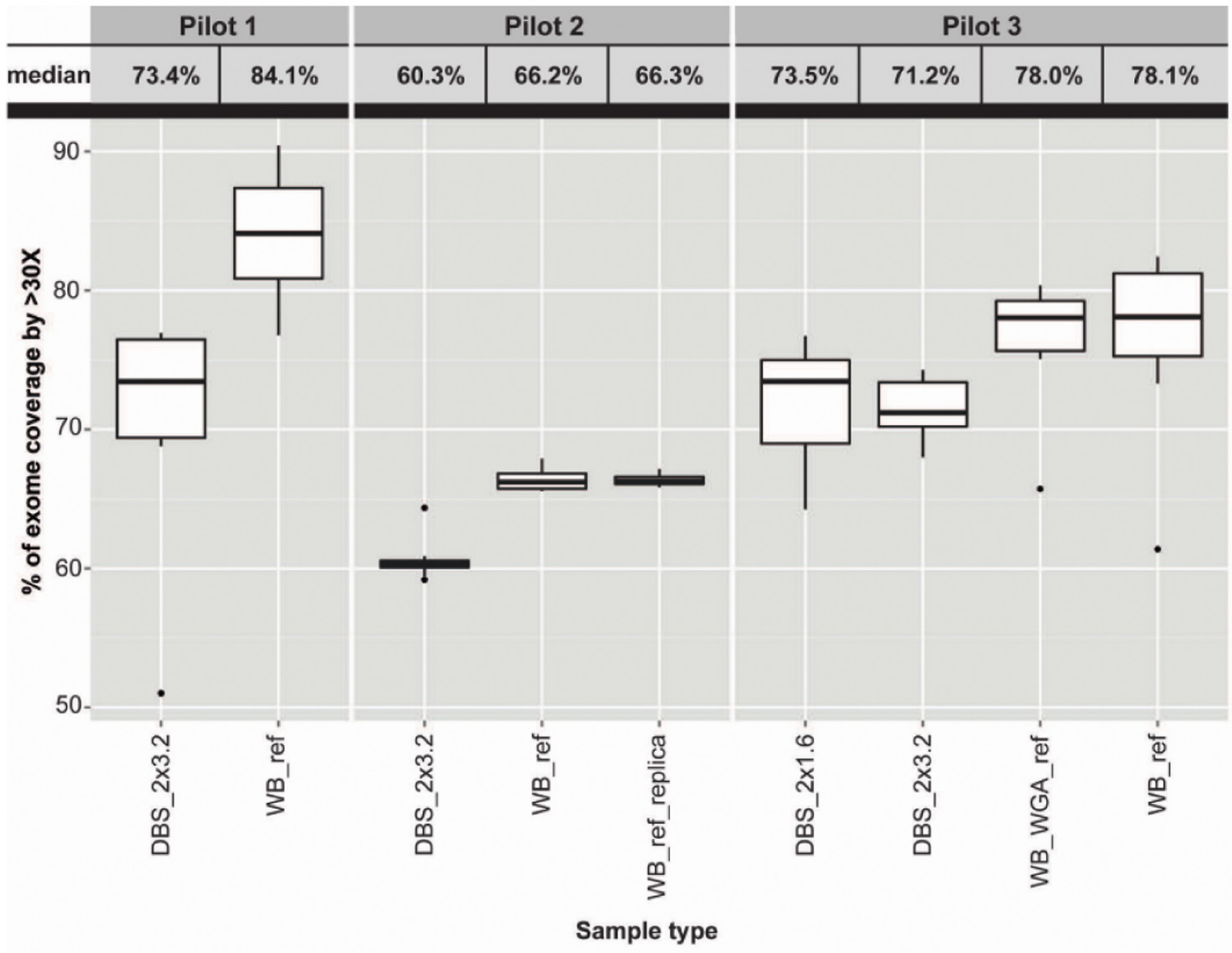
Exome coverage by depth. The data were presented with a box plot as percentage of exome coverage with sequencing depths greater than 30. The exact medians of the observations have been listed above the plot. From left to right, the pilots were depicted in the order: Pilot 1 with DBS_2x3.2 and WB_ref sample types, Pilot 2 with DBS_2x3.2, WB_ref and WB_ref replica sample types and Pilot 3 with DBS_2x1.6, DBS_2x3.2, WB_WGA_ref and WB_ref sample types, respectively. In the plot, the medians are given by a solid black line enclosed in boxes specifying the first and third quartiles. The whiskers represent the statistical dispersion of the data using the interquartile range (1.5*IQR). Data beyond 1.5*IQR range are outliers and plotted as dots. Note that the number of observations per sample type (not considering DBS_2x1.6, see below) equals the number of subjects included in the pilot, i.e. Pilot 1 = 7, Pilot 2 = 8 and Pilot 3 = 7. The DBS_2x1.6 sample type was plotted using all 21 observations, resulting from the triplicate experiments per subject of different sets of 2x1.6 mm discs included in Pilot 3. The coverage statistics were calculated with GATK using R to obtain the percentage of exome coverage per sample type shown.

Defining the required coverage is a subjective matter much depending on species, type of sample and scope of study. At a given site in a diploid genome, a depth threshold of 30X can be considered sufficient for calling high-quality variants. Defining this as our minimum threshold, we analysed the proportion of target regions in our data, displaying an average depth above 30X in each of the sample types (Fig 2). The statistics reveal a clear-cut difference in percentage of sequences reaching the standard threshold of 30X between the DBS and corresponding WB samples with the former exhibiting reduced threshold coverage. For the DBS_2x3.2 and WB_ref sample types, this difference was ~10.7% in Pilot 1, ~5.9% in Pilot 2 and ~6.9% in Pilot 3. Despite hereof, the results are still in an acceptable range for the DBS samples (between ~60–75% of exome coverage >30X) as compared with other samples for which high-quality WES data have been obtained [24, 25]. We also calculated the medians of percentage of exome coverage at depth >10X. For Pilot 1 this was 94.9% and 97.6% for the DBS_2x3.2 and WB_ref sample types, respectively. Pilot 2: 86.9%, 93.7% and 94.1% for the DBS_2x3.2, WB_ref and WB_ref_replica sample types, respectively. Pilot 3: 88.8%, 87.9%, 92.5% and 92.1% for the DBS_2x1.6, DBS_2x3.2, WB_WGA_ref and WB_ref sample types, respectively. At >10X, a reduced threshold coverage of ~2.7% in Pilot 1, ~6.8% in Pilot 2 and ~4.2% in Pilot 3 existed for the DBS_2x3.2 sample type as compared to the WB_ref sample type. We found no differences between the DBS_2x3.2 and DBS_2x1.6 sample types of Pilot 3, suggesting that coverage is independent of disc size in the range tested. In Pilot 3 in particular, the analyses have been run in triplicates, allowing us to perform a technical replication and verify the absence of random bias. We have not conducted further investigation into why the coverage is apparently reduced in WGA samples. Duplicate reads percentages was found to depend more on kit than on whether amplification was used. Respectively for pilot one/two/three Duplication rates for WB were 18%/9%/14%, for DBS they were 18%/9%/16%. The scope of this study was to investigate whether confidently discovered variant were reliable, we leave it to future studies to elucidate if genomic regions are lost. We conclude that the data quality is of a reasonable standard to proceed onwards launching the subsequent steps of variant calling.

### Variant Calling and Filtering

The variants were called by a multi-sample approach on a per-pilot basis, resulting in a single variant number count for each of the pilots. Variant calling was performed with merged HaplotypeCaller and UnifiedGenotyper calls as described in the methods section. In order to assess the performance of neonatal DBS samples as compared to WB samples, we needed to introduce a variant filter to remove the variability in genotype calling resulting from other factors, most commonly sequence quality. The multi-sample calling approach was chosen in order to focus our attention on the genotype call (i.e. the accuracy) rather than the emission variant site. In this way, while improving the calling of sites where a variant is present, we could identify specifically which parameters differentiate those loci where different genotypes (discordant) have been called. We thus explored the distribution of the parameters used by GATK Variant Quality Score Recalibration and found that discordant variants between sample types accumulated either at low sequencing depths (<20) or as variants exhibiting low call quality scores. Besides common genomic characteristics (variant-level filters) we also wanted to highlight characteristics of the samples, which might be responsible for discordant calls (sample-level filters). Therefore we adopted two criteria for the filtering: *i)* a variant-level filter on a generally recognised minimum threshold of sequence reliability, i.e. average sample depth >20 and *ii)* a sample-level filter summarising several sample-specific quality parameters, i.e. minimum genotype quality calculated only across WB_ref samples (minGQ_WB) >30. The first criterion functioned to remove the variants in areas covered by less than 20 reads (on average), and the second removed the variants of the WB_ref samples exhibiting genotype scores less than or equal to 30, while keeping those of high quality (>30) no matter the score of the corresponding variants in the other sample types. The variants were annotated for partitioning into SNPs, insertions, deletions and multiallelic calls, respectively. A summary of the variant calls before and after filtering is available in S1 Table. The calls were found to group by decreasing frequency of observation as SNPs > insertions > deletions > multiallelic calls with SNPs being greatly overrepresented in all pilots as well before as after filtering of the variants (84–94% of all variant calls are an SNP). As expected, the total number of variants varies depending on the capture strategy employed, which most likely result from actual differences in the genomic regions targeted including repetitive regions outside the exome. We subsequently used the variant calls for a more comprehensive comparison of sample types.

### Evaluation of Sample Performance by Pairwise Comparison of Genotypes—Concordance Rates

**Fig 3:**
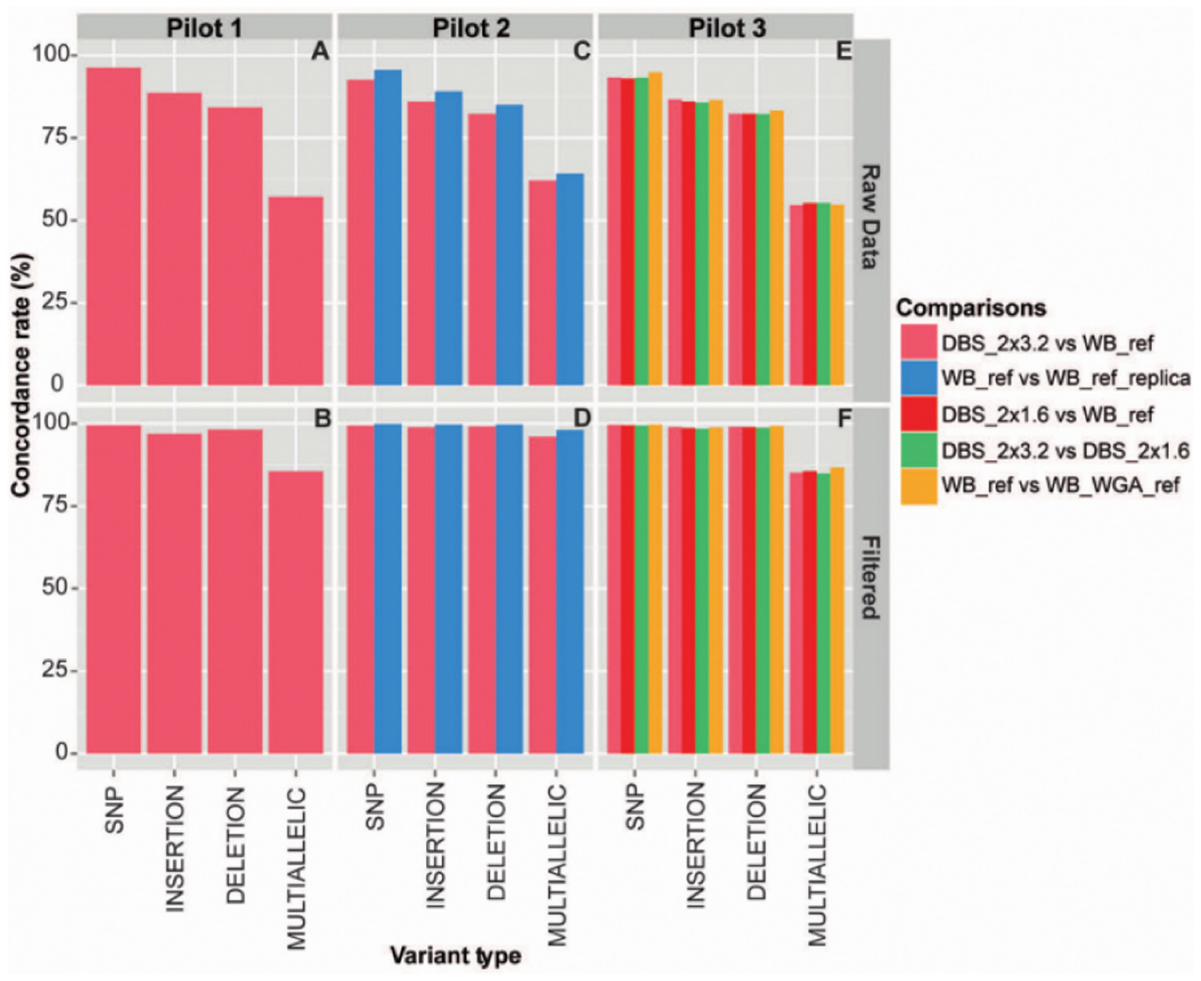
Comparison of sample types from variant calls—concordance rates. The concordance rates were calculated by pairwise comparison of variant calls before (upper panels) and after filtering (lower panels). The sample types compared were DBS_2x3.2 vs WB_ref in Pilot 1 **(A and B)**, DBS_2x3.2 vs WB_ref and WB_ref vs WB_ref replica in Pilot 2 **(C and D)** and DBS_2x1.6 vs WB_ref, DBS_2x3.2 vs WB_ref, DBS_2x3.2 vs DBS_2x1.6 and WB_ref vs WB_WGA_ref in Pilot 3 **(E and F)**. The rates have been presented per variant type: SNP, insertion, deletion and multiallelic calls, and comprise the averages of all comparisons made for a given sample pair, corresponding to the number of subjects in the pilot, i.e. Pilot 1 = 7, Pilot 2 = 8 and Pilot 3 = 7. Note that for comparisons using the DBS_2x1.6 sample type (see the fig), each individual replica was firstly compared to the WB_ref or DBS_2x3.2 sample types followed by the calculation of average values hereof, which were used in the figure.

To evaluate sample performance we executed a pairwise comparison of genotypes based on the variant calls resulting in a similarity measure between different sample types—the concordance rate (Fig 3). Concordance rates were obtained both before and after filtering of the calls and grouped by variant type into SNPs, insertions, deletions and multiallelic calls. The numbers supporting the graphs in Fig 3 are available in table format in S2 Table. We compared the DBS_2x3.2 and WB_ref sample types (all pilots) to obtain a measure of the feasibility of sequencing neonatal DBS samples. The WB_ref and WB_ref replica sample types of Pilot 2 were compared to establish a high-quality standard used for comparison to the DBS_2x3.2 vs WB_ref comparison, thereby testing the relative performance of the DBS_2x3.2 sample type. The DBS_2x1.6 vs WB_ref and DBS_2x3.2 vs DBS_2x1.6 comparisons of Pilot 3 were made in order to evaluate the potential of reducing the disc size, and the WB_ref vs WB_WGA_ref comparison for probing an effect resulting from the amplification reaction. A comparison of the DBS_2x1.6 sample type replicas with the WB_ref sample type, i.e. replica_1 vs WB_ref, replica_2 vs WB_ref and replica_3 vs WB_ref yielded similar concordance rates (see S1 Fig). Based on this, we decided to include all of the data of the DBS_2x1.6 sample type for calculation of the concordances displayed in Fig 3; that is the DBS_2x1.6 vs WB_ref and DBS_2x3.2 vs DBS_2x1.6 comparisons, respectively. This was done by initial comparison of each of the DBS_2x1.6 sample type replicas with the WB_ref or DBS_2x3.2 sample types, followed by the calculation of average values hereof. An initial look at the concordances in Fig 3 before filtering show strong dependency by variant type with SNPs > insertions > deletions > mulitiallelic calls, respectively (upper panels). This is similar to what has previously been observed in WES studies splitting calls by variant type, and results due to well-known difficulties of calling high-complexity variants as well as a general higher probability of encountering sequence variation between any two samples for variants of increasing lengths [26]. The pre-filtered concordance rates are consistent with other exome sequencing experiments dealing with sample replicas: previous reported rates were in the range of 82–92%, the numbers being largely dependent on the depth of coverage, as is also reported here [27]. Notably, we observe somewhat lower concordance rates of the DBS vs WB comparisons (i.e. all comparisons of neonatal DBS samples and WB reference samples) over the WB vs WB comparisons (all comparisons of WB reference samples) before filtering in Pilot 2 and Pilot 3, which may be ascribed to subtle differences in overall DNA quality of DBS-derived wgaDNA as compared to WB-derived DNA. This difference become neutralized after filtering yielding similar concordance rates between the DBS vs WB and WB vs WB comparisons independent of whether 2x3.2 mm or 2x1.6 mm discs were used for DNA extraction. Moreover, post-filtering the concordance rates of all pairwise compared samples are very close to 100% (the multiallelic calls of Pilots 1 and 3 omitted, for which rates only reaches 85–88%). This shows that DBS-derived wgaDNA no matter the input quantities, i.e. 2x3.2 mm or 2x1.6 mm discs can be set to perform similarly to high-quality DNA from a WB reference sample, if in case the proper filter settings are applied. This validates the use of DNA of neonatal DBSs for WES. The one most striking example illustrating the feasibility of sequencing the neonatal DNA can be inferred from the WB_ref vs WB_ref replica and DBS_2x3.2 vs WB_ref comparisons of Pilot 2. The fact that duplicate sequencing data produced using the same high-quality DNA (e.g. the WB_ref vs WB_ref replica comparison), scores similarly to that DNA set against wgaDNA of a neonatal DBS sample, proves the validity of the latter. One concern of using wgaDNA of neonatal DBS samples has been if the amplification reaction would introduce sequence errors and allele dropouts caused by the low inputs and presumed low quality of the DNA used. However, based on the very high similarities observed between the DBS vs WB and WB vs WB comparisons along with the very high concordances overall post-filtering, we conclude that this does not seem to constitute a problem.

### Methodological and Platform-Specific Considerations

Advantages, drawbacks and optimization potential exist for this methodology. Rare variant discovery for instance depends heavily on large cohort sizes, which is seldom available [28]. Biobanks like the DNSB representing an unbiased population subset and for which the samples can be linked to medical records may therefore be used. We suggest that the robustness of the current methodology be further addressed using a larger cohort, potentially hundreds of subjects to warrant validation in large research studies. One might also address the lower overall exome coverage observed for neonatal DBS samples compared to WB samples in this study. This may result from quality issues inherent to neonatal wgaDNA caused by either *i*) reduced quality of the input DNA used for WGA (i.e. fragmented and/or insufficient quantities), *ii*) amplification bias directed by the WGA kit used and/or *iii*) the fact that reduced quality of the input DNA promotes amplification bias depending on template-specific characteristics. The optimization could therefore be two-sided seeking to optimize the WGA reaction by testing different amplification kits, the time of amplification, the reaction volume and/or the number of sample replicates, or the quality of the input DNA could be improved for instance by enzymatic removal of surplus RNA and ssDNA to exclusively deliver dsDNA for the WGA reaction. Alternatively, one could try to completely omit WGA, and use raw DNA extract for library preparation. The DNA quantities will in this case be drastically reduced and incompatible with general input recommendations; therefore, this procedure will require extensive optimization. Finally, one could try to explore the potential of further downscaling the inputs of 2x1.6 mm discs for DNA extraction. This has additional prospects as Neonatal DBS are not the only sample type which yield minute amounts of DNA for subsequent analysis. Circulating tumour DNA and pre-implantation screening of oocytes are examples with similar limitations. While the approaches here could be applicable to pre-implantation screening, cancer genomes would need separate validation. An experiment comparing amplified and unamplified matched samples would allow exploration when the diploid assumption breaks down and polyploidy becomes a concern.

Advantages/Limitations exist for the technology itself compared with other high-throughput genotyping technologies. Rare variant detection by SNP microarray is for instance, delimited by probe design requiring hybridization of probes of known sequence: the current settings allow targeting only of shared variants common to a broader subset of the population [29]. In contrast, WES applies to all variants whether common or rare in the full gene set of an individual, providing highly reliable variant calls based on great sequencing depth and coverage. Compared to whole-genome sequencing (WGS), WES is more cost-effective, and because the majority of severe disease causing variants generally sites in protein-encoding genes; focusing only on this part (~1% of the genome) still provides a high yield of relevant information [30]. Nonetheless, WGS would no doubt stimulate a deeper and improved understanding of the genetic variation in intergenic regions including structural and non-coding variants associated with disease. We conclude that for exploration/identification of rare variants in regards to sample size and the ability to later on interpret the results, WES may currently be the most effective technology.

### Perspectives of the Methodology

Neonatal DBS samples may be used for WES in large cohort studies of disease, to identify the determinants underlying common and complex genetic diseases. Options also exist for inclusion of the methodology in the routine screening of neonates. Neonatal screening programmes generally rely on the quantification of metabolic markers with associated thresholds, followed by targeted DNA genotyping of whole-blood samples (or DBS samples) to establish the final diagnosis [3–7, 10]. The exploitation of complete WES in neonatal screening would indeed be faced with major political and ethical concerns relating to the use and storage safety of the data accumulated [31]. Patients and doctors would also be supplied with lots of sequence variation with no known function, or perhaps even with information about serious and untreatable diseases, which is a big ethical dilemma. Instead, one might look at a more targeted approach, scanning only those genes or mutations currently involved in neonatal screening programmes. This would certainly reduce the amount of excess extra data generated to an absolute minimum, and thus revoke less political and ethical objections/concerns. Overall, the outcome would be an improved setting, allowing the confirmation of metabolic markers at the genotypic level and vice versa. An alternative approach would be the establishment of an array-based panel of neonatal markers, which would provide an even more focused subset of information, however with the same possibilities as the aforementioned sequencing strategy for neonatal screening.

## Conclusions

The validation of samples by NGS typically involve the parallel sequencing of a high-quality DNA reference to be used as a gold standard for comparison. The array-genotyping of samples (or of high-quality reference DNA), is also frequently used for comparisons to the same samples tested on NGS platforms, providing a measure of sample reproducibility and/or of performance [32]. Here exome-replicas of high-quality DNA, i.e. WB vs WB comparisons were used in order to set a base-line value for evaluation of neonatal DBS samples for WES. The concordance rates of DBS vs WB and WB vs WB comparisons were in all cases similar and close to 100%, confirming that wgaDNA of neonatal DBS samples performs equally to high-quality reference DNA in WES. We found no effect of substituting 2x3.2 mm discs for 2x1.6 mm discs, allowing for a lot more flexibility in project designs relating to cohort size and/or the number of replicas. By this end, we have processed several sample types of 22 subjects allocated within three pilot studies, proving the feasibility of using neonatal DBS samples for WES. The reproducibility of WES was found to be satisfactory independent of provider and kit used. The integration hereof in research and/or in neonatal screening programmes remains to be seen.

## Supporting Information

S1 Fig Comparisons of the DBS_2x1.6 sample type replicas with the WB_ref sample type—concordance rates. The concordance rates were calculated by pairwise comparisons of the DBS_2x1.6 sample type replicas with the WB_ref sample type based on the variant calls sub-grouped according to variant type: SNPs, insertions, deletions and multiallelic calls. **(A)**. Graphic representation of the concordance rates before (upper panel) and after filtering (lower panel) of the calls with replica_1 vs WB_ref, replica_2 vs WB_ref and replica_3 vs WB_ref comparisons, respectively. **(B)**. The exact numbers of concordance (in %) entered in table form. Note the color-coding that has been used throughout with red, green and blue denoting the different comparisons. Each observation represents an average of seven comparisons, corresponding to the number of subjects in Pilot 3.
(PDF)

S1 Table Number and percentage of variant calls by variant type before and after filtering. The samples were assigned to variant calling on a per-pilot basis, and subsequently annotated by variant type into SNPs, insertions, deletions and multiallelic calls, respectively. The total number of variants and the relative percentages were determined both before and after filtering for **(A)** Pilot 1, **(B)** Pilot 2 and **(C)** Pilot 3. The criteria used for filtering were: *i)* average sample depth >20 and *ii)* minGQ_WB >30. Variant calling was with HaplotypeCaller and UnifiedGenotyper as described previously.
(PDF)

S2 Table Percentage (%) of concordance between sample types. The concordance rates were calculated by pairwise comparisons of variant calls sub-grouped by variant type (SNPs, insertions, deletions and multiallelic calls) before (Raw) and after filtering (Filt.). The sample types compared were DBS_2x3.2 vs WB_ref in Pilot 1, DBS_2x3.2 vs WB_ref and WB_ref vs WB_ref replica in Pilot 2 and DBS_2x3.2 vs WB_ref, DBS_2x1.6 vs WB_ref, DBS_2x3.2 vs DBS_2x1.6 and WB_ref vs WB_WGA_ref in Pilot 3. Note that the color-coding used matches the one used in Fig 3. Each number of concordance constitutes the average of all comparisons for a given sample pair, corresponding to the number of subjects in the pilot: Pilot 1 = 7, Pilot 2 = 8 and Pilot 3 = 7. For comparisons using the DBS_2x1.6 sample type (see the table), each individual replica was firstly compared to the WB_ref or DBS_2x3.2 sample types followed by the calculation of average values hereof, which were used in the table.
(PDF)

## Abbreviations

DNSB: Danish Neonatal Screening Biobank
DBS: Dried blood spot
NGS: Next-generation sequencing
SNP: Single-nucleotide polymorphism
WGA: Whole-genome amplification
WGS: Whole-genome sequencing
WES: Exome-targeted next-generation sequencing
WB: Whole-blood
wgaDNA: Whole-genome amplified DNA
