# Supplementary material for: High-Quality Exome Sequencing of Whole-Genome Amplified Neonatal Dried Blood Spot DNA
Source: PLoS One. 2016 Apr 18;11(4):e0153253. doi: 10.1371/journal.pone.0153253 (PMC4835089; doi:10.1371/journal.pone.0153253)
Supplement: S1 Table — The samples were assigned to variant calling on a per-pilot basis, and subsequently annotated by variant type into SNPs, insertions, deletions and multiallelic calls, respectively. The total number of variants and the relative percentages were determined both before and after filtering for (A) Pilot 1, (B) Pilot 2 and (C) Pilot 3. The criteria used for filtering were: i) average sample depth >20 and ii) minGQ_WB >30. Variant calling was with HaplotypeCaller and UnifiedGenotyper as described previously. (PDF) [file pone.0153253.s002.pdf]

A

	Pilot 1			
	Raw Data		Filtered	
	Numbers	% of total	Numbers	% of total
SNP	136,915	84.4	99,244	90.1
INSERTION	11,457	7.1	6,222	5.6
DELETION	9,625	5.9	4,177	3.8
MULTIALLELIC	4,135	2.6	550	0.5
Total	162,132	100.0	110,193	100.0

B

	Pilot 2			
	Raw Data		Filtered	
	Numbers	% of total	Numbers	% of total
SNP	155,755	88.0	86,540	93.0
INSERTION	8,063	4.6	2,870	3.1
DELETION	10,423	5.9	3,456	3.7
MULTIALLELIC	2,838	1.6	214	0.2
Total	177,079	100.0	93,080	100.0

C

	Pilot 3			
	Raw Data		Filtered	
	Numbers	% of total	Numbers	% of total
SNP	128,720	87.6	79,285	93.7
INSERTION	6,214	4.2	2,374	2.8
DELETION	8,191	5.6	2,723	3.2
MULTIALLELIC	3,772	2.6	210	0.2
Total	146,897	100.0	84,592	100.0
