# Supplementary material for: High-Quality Exome Sequencing of Whole-Genome Amplified Neonatal Dried Blood Spot DNA
Source: PLoS One. 2016 Apr 18;11(4):e0153253. doi: 10.1371/journal.pone.0153253 (PMC4835089; doi:10.1371/journal.pone.0153253)
Supplement: S2 Table — The concordance rates were calculated by pairwise comparisons of variant calls sub-grouped by variant type (SNPs, insertions, deletions and multiallelic calls) before (Raw) and after filtering (Filt.). The sample types compared were DBS_2x3.2 vs WB_ref in Pilot 1, DBS_2x3.2 vs WB_ref and WB_ref vs WB_ref replica in Pilot 2 and DBS_2x3.2 vs WB_ref, DBS_2x1.6 vs WB_ref, DBS_2x3.2 vs DBS_2x1.6 and WB_ref vs WB_WGA_ref in Pilot 3. Note that the color-coding used matches the one used in Fig 3. Each number of concordance constitutes the average of all comparisons for a given sample pair, corresponding to the number of subjects in the pilot: Pilot 1 = 7, Pilot 2 = 8 and Pilot 3 = 7. For comparisons using the DBS_2x1.6 sample type (see the table), each individual replica was firstly compared to the WB_ref or DBS_2x3.2 sample types followed by the calculation of average values hereof, which were used in the table. (PDF) [file pone.0153253.s003.pdf]

	Pilot 1		Pilot 2				Pilot 3							
	Raw	Filt.	Raw	Filt.	Raw	Filt.	Raw	Filt.	Raw	Filt.	Raw	Filt.	Raw	Filt.
SNP	96.4	99.5	92.6	99.4	95.6	99.9	93.4	99.6	93.0	99.5	93.2	99.5	94.9	99.7
INSERTION	88.7	97.0	86.0	99.0	89.1	99.7	86.7	99.0	86.0	98.7	85.7	98.4	86.6	98.9
DELETION	84.3	98.2	82.3	99.2	85.0	99.8	82.3	99.1	82.4	99.0	82.3	98.7	83.3	99.3
MULTIALLELIC	57.2	85.6	62.1	96.0	64.2	98.1	54.7	85.2	55.3	85.6	55.3	84.9	54.8	86.7
Comparisons	DBS_2x3.2 vs WB_ref		DBS_2x3.2 vs WB_ref		WB_ref vs WB_ref_rep.		DBS_2x3.2 vs WB_ref		DBS_2x1.6 vs WB_ref		DBS_2x3.2 vs DBS_2x1.6		WB_ref vs WB_WGA_ref	
