# Supplementary material for: High-Quality Exome Sequencing of Whole-Genome Amplified Neonatal Dried Blood Spot DNA
Source: PLoS One. 2016 Apr 18;11(4):e0153253. doi: 10.1371/journal.pone.0153253 (PMC4835089; doi:10.1371/journal.pone.0153253)
Supplement: S1 Fig — The concordance rates were calculated by pairwise comparisons of the DBS_2x1.6 sample type replicas with the WB_ref sample type based on the variant calls sub-grouped according to variant type: SNPs, insertions, deletions and multiallelic calls. (A). Graphic representation of the concordance rates before (upper panel) and after filtering (lower panel) of the calls with replica_1 vs WB_ref, replica_2 vs WB_ref and replica_3 vs WB_ref comparisons, respectively. (B). The exact numbers of concordance (in %) entered in table form. Note the color-coding that has been used throughout with red, green and blue denoting the different comparisons. Each observation represents an average of seven comparisons, corresponding to the number of subjects in Pilot 3. (PDF) [file pone.0153253.s001.pdf]

A

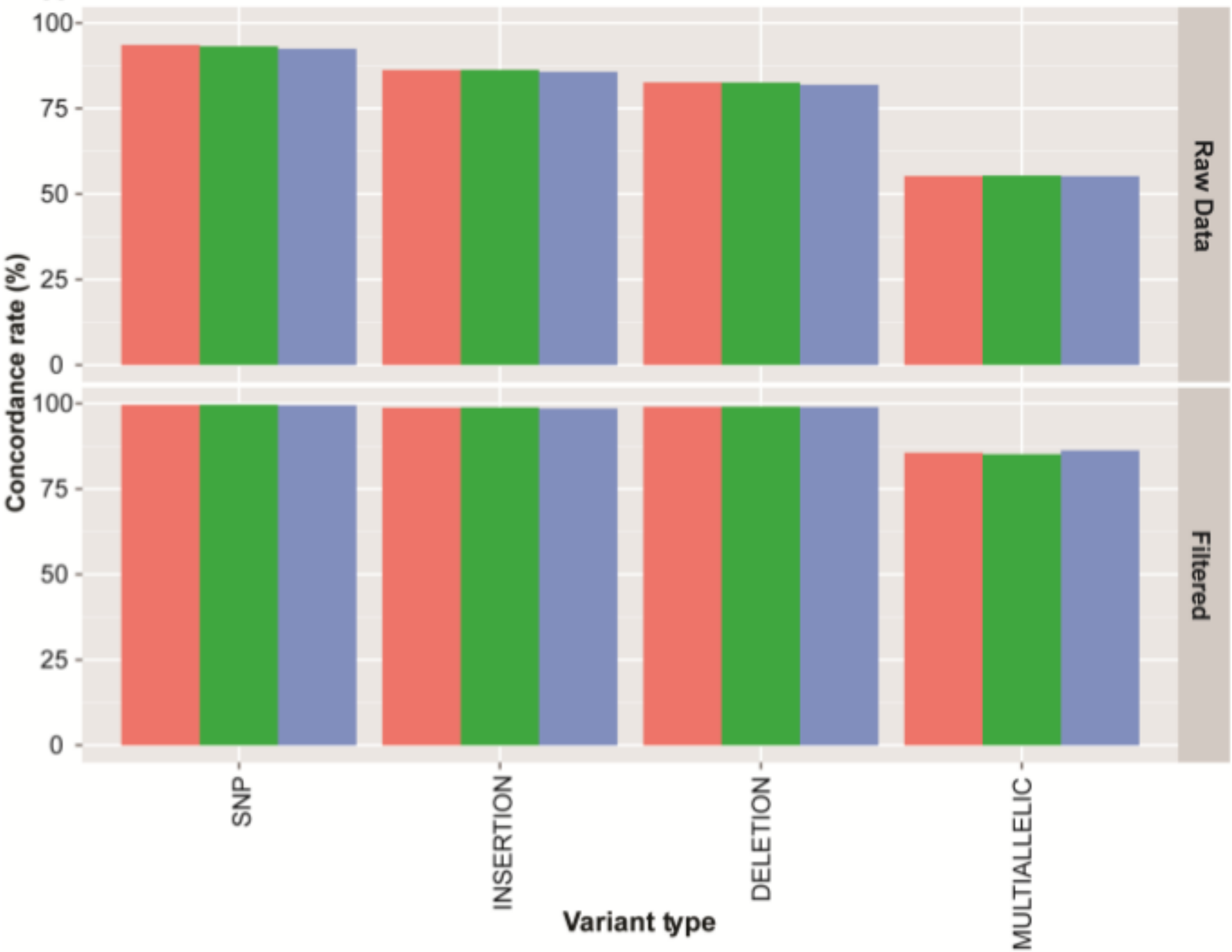

B

	SNP			INSERTION			DELETION			MULTIALLELIC		
**Raw Data**	93.6	93.2	92.4	86.2	86.2	85.7	82.6	82.6	81.9	55.2	55.3	55.2
**Filtered**	99.6	99.6	99.4	98.7	98.8	98.5	99.0	99.0	98.9	85.5	85.1	86.2

Variant type

Comparisons replica_1 vs WB_ref | replica_2 vs WB_ref | replica_3 vs WB_ref
